# Family History Information Extraction With Neural Attention and an Enhanced Relation-Side Scheme: Algorithm Development and Validation

**DOI:** 10.2196/21750

**Published:** 2020-12-01

**Authors:** Hong-Jie Dai, You-Qian Lee, Chandini Nekkantti, Jitendra Jonnagaddala

**Affiliations:** 1 College of Electrical Engineering and Computer Science Department of Electrical Engineering National Kaohsiung University of Science and Technology Kaohsiung City Taiwan; 2 School of Post-Baccalaureate Medicine Kaohsiung Medical University Kaohsiung Taiwan; 3 National Institute of Cancer Research National Health Research Institutes Tainan Taiwan; 4 CGD Health Pty Ltd Belconnen Australia; 5 School of Public Health and Community Medicine University of New South Wales Sydney Australia

**Keywords:** family history information, natural language processing, deep learning, electronic health record

## Abstract

**Background:**

Identifying and extracting family history information (FHI) from clinical reports are significant for recognizing disease susceptibility. However, FHI is usually described in a narrative manner within patients’ electronic health records, which requires the application of natural language processing technologies to automatically extract such information to provide more comprehensive patient-centered information to physicians.

**Objective:**

This study aimed to overcome the 2 main challenges observed in previous research focusing on FHI extraction. One is the requirement to develop postprocessing rules to infer the member and side information of family mentions. The other is to efficiently utilize intrasentence and intersentence information to assist FHI extraction.

**Methods:**

We formulated the task as a sequential labeling problem and propose an enhanced relation-side scheme that encodes the required family member properties to not only eliminate the need for postprocessing rules but also relieve the insufficient training instance issues. Moreover, an attention-based neural network structure was proposed to exploit cross-sentence information to identify FHI and its attributes requiring cross-sentence inference.

**Results:**

The dataset released by the 2019 n2c2/OHNLP family history extraction task was used to evaluate the performance of the proposed methods. We started by comparing the performance of the traditional neural sequence models with the ordinary scheme and enhanced scheme. Next, we studied the effectiveness of the proposed attention-enhanced neural networks by comparing their performance with that of the traditional networks. It was observed that, with the enhanced scheme, the recall of the neural network can be improved, leading to an increase in the F score of 0.024. The proposed neural attention mechanism enhanced both the recall and precision and resulted in an improved F score of 0.807, which was ranked fourth in the shared task.

**Conclusions:**

We presented an attention-based neural network along with an enhanced tag scheme that enables the neural network model to learn and interpret the implicit relationship and side information of the recognized family members across sentences without relying on heuristic rules.

## Introduction

Family history information (FHI), such as a patient’s family members and their corresponding side of the family (ie, maternal or paternal), health-related problems like medical histories and current disorders, and habits of substance use, is not only an essential risk factor for many chronic and hereditary diseases such as cardiovascular diseases, diabetes, and cancers [1] but also an important clue for individualized disease diagnosis, treatment, prediction, and prevention [2-6]. FHI is usually described in an unstructured free-text format within a patient’s electronic health record, and its content depends on pieces of information provided by patients about the health situation of their relatives during clinical visits. Therefore, it will be beneficial if natural language processing (NLP) can be employed to identify FHI to provide a more comprehensive view of patient-centered information for physicians.

In general, FHI consists of 3 essential factors, including the relationship between family members, side of the members, and associated observations. Early studies working on the identification of FHI [7,8] relied on the Unified Medical Language System to extract FHI and applied rules to associate the relations. The release of available FHI training corpora such as the BioCreative/OHNLP challenge 2018 [9] and the 2019 n2c2/OHNLP shared tasks prompted the advancement of NLP for automatically extracting FHI. Researchers currently apply a variety of approaches to tackle the task of FHI extraction. For example, Dai [10] introduced 3 inside, outside, beginning (IOB)2-based tag sets that can be utilized to identify family members and their observations along with the bidirectional long short-term memory (BiLSTM)-conditional random field (CRF) model. The first was the standard IOB-2 scheme, which only captures the spans of the mentioned family members and observations. Therefore, 5 tags including B/I-FM, B/I-Ob, and O were used. The second scheme further encodes the family side information in the tag set for family members. For example, “Mother” is not associated with any family side values, so its mention is assigned with the B/I-FM-NA tag, while other tag sets include the B/I-FM-Paternal and B/I-FM-Maternal tags. The relation-side scheme was the last proposed tag scheme in which both the type and side properties are encoded. Consequently, all possible combinations of the 2 properties that appeared in the training set were represented by the tag scheme.

Without encoding both the side and relationship information in tag sets like the relation-side scheme for model training, previous work had to develop sophisticated postprocessing rules that relied on commonsense knowledge and surrounding text to infer the 2 properties of family members and integrate handcrafted rules with deep learning models in a pipeline structure. In addition to the challenge of optimizing both submodules separately, there are at least two other known limitations of applying postprocessing rules. One is the inability to determine cases like indirect relatives as pointed out by Dai [10] and Shi et al [11], and the other is the general ability to classify FHIs represented in different writing styles. Unfortunately, although the aforementioned relation-side scheme is expected to facilitate the development of a single end-to-end model to conquer the task of FHI extraction, the experiment results by Dai [10] revealed issues of insufficient and imbalanced training instances. In light of these constraints, we eliminated the postprocessing rules and managed the issue of training instances by proposing an enhanced relation-side tag scheme. Moreover, we introduced the attention-based neural network structure to better exploit intrasentence and intersentence information to determine the FHIs requiring cross-sentence inference.

## Methods

We preprocessed medical notes to generate sentences and the corresponding tokens associated with their part-of-speech information via our clinical toolkit [12]. By formulating the FHI extraction task as a sequential labelling problem, we applied the proposed tag scheme to encode the gold annotations to generate the datasets for training the proposed network models. In the following subsections, we first introduce the relation-side scheme proposed by Dai [10] and the enhanced version proposed in this work, followed by descriptions of the architecture of the developed model that can utilize cross-sentence information via the sentence-level and document-level neural attentions.

### Tag Scheme Design

In order to exclude the need for postprocessing steps, Dai [10] presented the relation-side scheme in which both the side and family relationship properties are encoded within the IOB tag sets for family member entities. Table 1 displays an example of the encoded annotations. Taking the first family member mention “two paternal aunts” as an example, we included the side and relationship information (“paternal” and “aunt,” respectively, in this case) in the tag set. Since both side and relationship attributes were encoded and later learned by the machine learning model, it is not necessary to apply postprocessing algorithms to infer the 2 properties.

**Table 1:** An example sentence encoded with the relation-side scheme and enhanced version: “The patient has two paternal aunts and one paternal half–brother, all were diagnosed with type-2 diabetes.”

Word	Relation-side scheme	Enhanced relation-side scheme
has	O	O
two	B-Aunt-Paternal	I-FM
paternal	I-Aunt-Paternal	I-FM
aunts	I-Aunt-Paternal	E-Aunt-Paternal
and	O	O
one	B-Brother-NA	I-FM
paternal	I-Brother-NA	I-FM
half-brother	I-Brother-NA	E-Brother-NA
,	O	O
…	…	…
type-2	B-OB	B-OB
diabetes	I-OB	I-OB

The drawback of the relation-side scheme is that the tag scheme combines all required information in its encoding, which is too specific and may result in problems of insufficient training instances. Take the annotations of the n2c2/OHNLP shared task as an example. In their annotations, the first-degree relatives, which include 8 types of family members (ie, Father, Mother, Parent, Sister, Brother, Daughter, Son, and Child), do not have the value of the family side property (refer to the tags ending with “NA” in Table 1). However, annotations of the other 7 family members (ie, Grandmother, Grandfather, Grandparent, Cousin, Sibling, Aunt, and Uncle) contain both properties. Therefore, we have at most 8 x 2 x 1 + 7 x 2 x 3 = 58 tags for family members. Consequently, we proposed the enhanced relation-side scheme in which only the I (inner) and E (end) tags were used and the relationship and side properties were only encoded in the E tag. For example, in Table 1, we can see that the word “paternal” of the 2 family member mentions was encoded by I-FM, which implies that the word is a part of a family mention. The annotations for the last words of the 2 mentions were encoded by including their relationship and side information. The number of possible tags was reduced to 1 + 8 x 1 + 7 x 3 = 30. On the other hand, for observations like “type-2 diabetes” in Table 1, both schemes used the ordinary IOB tag set to encode the annotations. The enhanced tag scheme is preferred because it greatly reduced the size of the tag sets and transition matrix used later in the CRF layer of the developed model.

### Baseline Network Architecture

We used the network architecture developed by Dai [10] as a baseline. The network architecture is very similar to the entity recognition part of the network developed by Shi et al [11], with the major difference being that the latter further extended the network with an additional BiLSTM to create a joint learning model. Both were top-ranked systems in the BioCreative/OHNLP challenge.

In our implementation, the baseline architecture consists of 2 core parts, with the first being the representation layer in which the sequence of tokens **t** = {*t*_1_,*t*_2_,…,*t*_n_} was represented as a vector by concatenating the character-level representation based on convolutional neural networks, pre-trained word representations, the randomly initialized part-of-speech embedding, and the pre-trained Unified Medical Language System embedding [13]. Based on the investigation by Dai [10] on the effectiveness of applying different pretrained word embeddings to the task of FHI extraction and the effectiveness of the recent advancement of contextualized word representations, global vectors for word representation (GloVe) [14] and the embeddings from language models (ELMo) [15] were used to represent the tokens. The concatenated representation was then inputted to a BiLSTM network with CRF as the output layer to infer predictions for each token.

The BiLSTM CRF networks have been shown to be able to efficiently model contextual information and label dependencies [16] and is currently a strong baseline. However, one major constraint is that the networks can only exploit contexts within individual sequences but cannot digest cross-sentence information. To overcome this limitation, we enhanced the baseline model by introducing the neural attentions described in the next subsection.

### Attention-Enhanced BiLSTM-CRF Network Architecture

**Figure 1:**
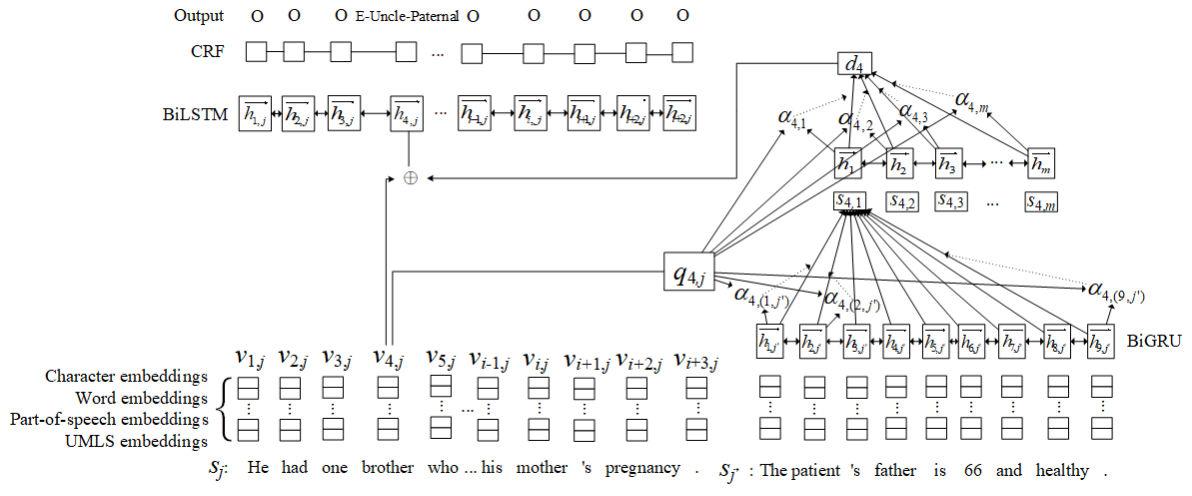
Proposed attention-enhanced bidirectional long short-term memory (BiLSTM)-conditional random field (CRF) network architecture. ⊕ indicates a concatenation of two vectors. BiGRU: bidirectional gated recurrent unit; UMLS: Unified Medical Language System.

Figure 1 illustrates the network architecture of the proposed attention-enhanced network. In the network, for each token *t_i,j_* in a given sentence *s_j_*, we applied the attention mechanism to make it attend to certain tokens among all sentences {*s*_1_,*s*_2_,…,*s*_m_} of the document **d** to allow the model to determine the type and the attributes of the token *t_i,j_* by considering information at the sentence and document levels. Each sentence *s_j_* in the input document **d** is expressed as **t*_j_*** = {*t*_1,_*_j_*,*t*_2,_*_j_*,…,*t_n,j_*} where *n* is the number of tokens in *s_j_*.

Like our baseline model, each token *t_i,j_* in the sequence of tokens **t*_j_*** was represented as a vector *v_i,j_* by concatenating the embeddings described in the previous subsection. Before sending the vector to the BiLSTM-CRF layer as an input, a hierarchical attention layer is introduced to enrich the vector to enable the model in utilizing cross-sentence information. In the attention layer, the attention score, which conveys the associations between the current token’s representation *v_i,j_* and all tokens’ representations in **d**, was hierarchically calculated using the following content-based function adapted from Luong et al [17] where **W*_t_*** and **W*_t’_*** are learned parameters and *h_i’,j’_* is the hidden state of the bidirectional gated recurrent unit at the token *t_i’,j’_* from another sentence:

*s_j’_*: *q*(*v_i,j_*) = **W*_t_****v_i,j_* + *b_q_***(1)**

*t_w*(*h_i’,j’_*) = tanh(**W*_t’_****h_i’,j’_* + *b_t_s_*) **(2)**

The score was calculated sentence-wise for the token *t_i,j_* to derive its attention weight *α_i,(i’,j’)_* for the token *t_i’,j’_* in the sentence *s_j’_*:

score(*v_i,j_*,*h_i’,j’_*) = *q*(*v_i,j_*)^T^*t_w*(*h_i’,j’_*) **(3)**

The aggregated score s*_i,j’_* for all tokens in *s_j’_* was calculated as follows:


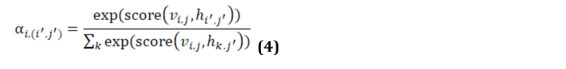



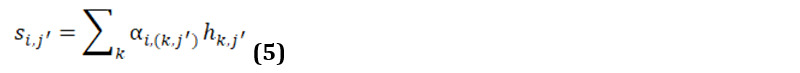


Given the aggregated sentence scores **s***_i_* = {*s_i,_*_1_,*s_i,_*_2_,…*s_i,m_*} for the token *t_i,j_*, we derived a document vector *d_i_* in a similar way to summarize the information from all sentences. First, a bidirectional gated recurrent unit was used to encode **s***_i_*, which can generate the hidden state *h_k_* for the *k*^th^ vector in **s***_i_*. Analogous to the hierarchical attention networks proposed by Yang et al [18], we rewarded sentences that provide clues to infer the type and attribute information of the target token *t_i,j_* using the following attention mechanism:

*t_s*(*h_k_*) = tanh(**W***_s_h_k_* + *b_t_s_*) **(6)**

score(*v_i,j_*,*h_k_*) = *q*(*v_i,j_*)^T^*t_s*(*h_k_*) **(7)**

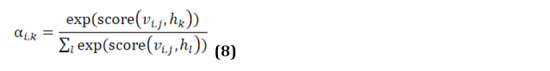



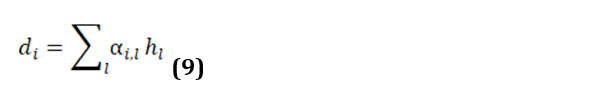


The output of the hierarchical attention layer *d_i_* can be considered as a document-level vector that summarizes information across sentences in **d** for token *t_i,j_*, which provides clues for determining FHI. Finally, the document vector was treated as an additional feature vector and concatenated with the original token representations to form the input of the BiLSTM-CRF model.

### Experiment Configurations

The dataset released by the 2019 n2c2/OHNLP shared task was used to evaluate the performance of the proposed network architecture along with the designed tag scheme. The training and test sets consist of 99 and 117 unstructured clinical notes, respectively. We randomly selected 83 of the 99 notes as the final training set, with the remaining 16 notes as the validation set in the training process. The validation set was not used in training but was used to determine the optimum parameters without overfitting the training set. We configured 3 runs for the participation of the n2c2/OHNLP family history extraction track. Both the first and second configurations were based on the proposed neural attention network along with the enhanced relation-side scheme. The only difference is that when processing a given sentence, the first configuration took all sentences in the note into consideration, while the second only examined sentences before the current one. The last run was based on the baseline BiLSTM-CRF network described in the previous subsection.

In addition to the submitted runs, we studied the effectiveness of the proposed tag scheme by training the baseline and attention-enhanced networks with different schemas and reported their performance on the test set. Table 2 summarizes all the configurations studied in this work. All the networks were implemented using CUDA 10.1 and PyTorch libraries trained on machines equipped with NVIDIA Tesla P100 graphics cards. The mini-batch gradient descent along with Adam [19] was used for optimizing the parameters. The epoch was set to 200, and the early stopping strategy (a patience value of 50) was used if no improvement in the F score or loss was observed or the loss became zero on the validation set. The same set of hyperparameters and a fixed random seed were used to train all the configurations shown in Table 2.

**Table 2:** Summary of the configurations studied in this work.

Configuration	Description	Notation
Baseline + relation-side scheme	BiLSTM-CRF^a^ with relation-side scheme	B-RS
Baseline + enhanced relation-side scheme	BiLSTM-CRF with enhanced relation-side scheme	B-ERS
Attention + relation-side scheme	Attention-enhanced BiLSTM-CRF with relation-side scheme	A-RS
Attention + enhanced relation-side scheme	Attention-enhanced BiLSTM-CRF with enhanced relation-side scheme paying attention to limited sentences	A-ERS
Attention + enhanced relation-side scheme (+)	Attention-enhanced BiLSTM-CRF with enhanced relation-side scheme paying attention to all sentences	A-ERS+

^a^BiLSTM-CRF: bidirectional long short-term memory-conditional random field.

The official evaluation script [20] released by the organizers was used to report the performance of the developed models. The performance for the recognized family member mentions including their family side attributes and observations were reported in terms of the standard precision (P), recall (R), and F1-measure (F) defined as follows at the article level:

Precision = TP/TP + FP **(10)**

Recall = TP/TP + FN **(11)**

F_1_ = 2 x P x R/(P + R) **(12)**

For each recognized family member mention, the 15 types of relatives described in the previous subsections were considered for evaluation. For each correctly recognized family member mention, its side of the family (ie, paternal, maternal, or not available) must also be correctly classified so that a true positive can be counted, else both the false positive and false negative are increased by one.

## Results

In the following subsections, we first compare the performance of the baseline model with the enhanced relation-side scheme to that of the model with the original scheme. Subsequently, we investigate the effect of the proposed attention-enhanced network architectures.

### Effect of the Enhanced Relation-Side Scheme

Table 3 outlines the performance of the baseline models with the original relation-side scheme (B-RS) and the proposed enhanced version (B-ERS). The last column of the table also shows the F scores for both models on the validation set and the number of executed epochs before terminating. With the early stopping strategy described in the previous section, both models terminated their training phase in advance and achieved F scores larger than 0.94 on the training set. The B-ERS model generally outperformed the B-RS model on the validation and test sets. It can be observed that the recalls of the B-ERS model for both family member mention and observation were better than those of the B-RS model by 0.061 and 0.117, respectively, which led to an increase in the overall F score of 0.024. These results demonstrated that the proposed enhanced scheme provides a better representation and facilitates a better learning process for the model.

**Table 3:** Effect of the proposed enhanced relation-side scheme on the test and validation sets.

Configuration	Family member			Observation			Overall	F on the validation set	Number of epochs
	P^a^	R^b^	F	P	R	F	F		
B-RS^c^	0.896	0.658	0.759	0.718	0.813	0.762	0.761	0.795	88
B-ERS^d^	0.882	0.719	0.792	0.674	0.928	0.781	0.785	0.822	124

^a^P: precision.

^b^R: recall.

^c^B-RS: bidirectional long short-term memory-conditional random field with relation-side scheme.

^d^B-ERS: bidirectional long short-term memory-conditional random field with enhanced relation-side scheme.

### Effect of the Cross-Sentence Attention

Table 4 provides the results of the comparative evaluation in accordance with the P, R, and F scores of the B-RS model. All proposed attention-enhanced BiLSTM-CRF models obtained better P, R, and F scores than those of the baseline model (B-RS). Among them, A-ERS+, our best submitted run during the 2019 n2c2/OHNLP shared task, had the best performance with improvements of 0.034, 0.058, and 0.046 in terms of P, R, and F scores, respectively. It is noted that the proposed attention mechanism apparently improved the recall of family member mention for all 3 models. In particular, the recall of A-ERS+ can be boosted by 0.118, resulting in a better F score of 0.807. Furthermore, the F scores of observations among the attention-enhanced models were also improved by at least 0.022.

**Table 4:** Comparison of the performance of the different attention-enhanced bidirectional long short-term memory-conditional random field (BiLSTM-CRF) models.

Performance measures		A-RS^a^	A-ERS^b^	A-ERS+^c^
**Family member**				
	Precision	–0.031	–0.008	–0.046
	Recall	+0.053	+0.092	+0.118
	F score	+0.022	+0.054	+0.052
**Observation**				
	Precision	–0.031	+0.011	+0.061
	Recall	+0.053	+0.074	+0.018
	F score	+0.022	+0.038	+0.042
Overall F score		+0.007	+0.044	+0.046

^a^A-RS: attention-enhanced BiLSTM-CRF with relation-side scheme.

^b^A-ERS: attention-enhanced BiLSTM-CRF with enhanced relation-side scheme paying attention to limited sentences.

^c^A-ERS+: attention-enhanced BiLSTM-CRF with enhanced relation-side scheme paying attention to all sentences.

## Discussion

### Principal Findings

Dai [10] provided an intensive analysis of the effectiveness of applying different tag schemes to the task of FHI extraction. In short, the advantage of applying the relation-side scheme is that we can eliminate the creation of heuristic rules for determining the relationship and side information of the recognized family member mentions, which is a major issue experienced by using standard tag schemes. Nevertheless, Dai [10] also pointed out that employing the scheme could lead to sparse and imbalanced training instances if the released dataset was small, which hinders the construction of a reliable model for identifying the desired properties of recognized mentions.

In this study, we addressed these issues by developing an enhanced relation-side scheme that achieved promising results, as shown in Table 4. We believe that the performance gain comes from the refined tag set distribution, where the enhanced scheme has significantly fewer tag types (30 vs 66). The tag with the highest distribution in the enhanced scheme is I-FM, which indicates that 35% of family member mentions in the training set consist of more than 1 token after tokenization, followed by E-FM-Mother-Na (7%), E-FM-Sister-NA (6%), E-FM-Father-NA (6%), E-FM-Brother-NA (6%), E-FM-Aunt-Maternal (5%), E-FM-Son-NA (4%), E-FM-Aunt-Paternal (4%), E-FM-Daughter-NA (3%), and E-FM-Uncle-Paternal (3%; Multimedia Appendix 1).

By contrast, no tags occupied more than 10% of the overall distribution in the original relation-side scheme. The top 10 tag types are as follows: B-FM-Mother-NA (7%), B-FM-Father-NA (6%), B-FM-Sister-NA (6%), B-FM-Brother-NA (5%), B-FM-Aunt-Maternal (5%), I-FM-Aunt-Maternal (4%), B-FM-Son-NA (4%), B-FM-Aunt-Paternal (4%), B-FM-Daughter-NA (4%), and I-FM-Grandmother-Maternal (3%; Multimedia Appendix 1). It is also worth noting that some family member types possessed frequent inner tags. For example, there are more instances of the inner tag for “Aunt-Maternal” (I-FM-Aunt-Maternal) than other members such as son and daughter, and the inner tag of “Grandmother-Maternal” (I-FM-Grandmother-Maternal) appears more frequently than its beginning tag. A scrutiny of the example shown in Table 1 revealed that the use of the tag scheme increased the degree of lexical ambiguity. For instance, the word “paternal” in Table 1 is assigned with 2 different tags (“I-Brother-NA” and “I-Aunt-Paternal”) although it is just a hint for the mention of family members. This observation also leads to the issue of imbalanced training samples because the word “paternal” could be a beginning or inner word for several types of family members. However, the distribution of those member types is skewed in the training set.

On the other hand, the enhanced relation-side scheme uses I-FM to capture clues that enable the model to learn and make final classifications based on the word with the most informative representation, which is usually the last word in terms of the family member entities. The scheme also resolves the problem of insufficient training samples. By considering Table 1 as an example, the traditional IOB2 scheme encodes all properties in its tag set. As a result, the token “aunts” can be associated with 6 different kinds of tags (B/I-Aunt-Paternal/Maternal/NA). With respect to the enhanced scheme, the token can only be associated with one of the E-Aunt-Paternal/Materal/NA tags, regardless of it being a single or compound noun. Examination of this problem from a different perspective is displayed in Table 5, which shows an evidently higher level of ambiguity in the relation scheme against the enhanced version. It was also found that even with the final CRF layer, the model with the original relation-side scheme could generate illegal tag sequences in the decoding phase, for instance a B-Aunt-Paternal followed by an I-Brother-Paternal, which was not observed in the model with the enhanced scheme.

**Table 5:** Comparison of the degrees of ambiguity between the relation-side scheme and enhanced relation-side scheme. Note that the tokens that were only associated with the “O” tag were excluded.

Scheme type	Number of possible tags associated with a token											
	1	2	3	4	5	6	7	8	9	10	17	20
Relation-side scheme	535	174	41	3	3	8	5	1	5	1	1	1
Enhanced relation-side scheme	535	188	38	11	5	1	0	0	0	0	0	0

Another challenge that was brought up in Dai [10] is that the perception of the member type and its side property may require cross-sentence inference. In light of this issue, we proposed using the attention mechanism to enhance the ability of the model for identifying these 2 properties. As shown in Table 4, the F scores of not only the family members but also the observations were improved by implementing the attention mechanism, with the improvement particularly due to a boost in the recall. After comparing the results of the models with and without the attention mechanism, we confirmed that the attention-enhanced networks can better exploit the intrasentence and intersentence information to successfully determine the type and side information of family member mentions in which the traditional model failed. Take the following 2 sentences as an example:

The **father** of the baby has a **maternal uncle** with a repaired cleft lip. His **uncle** is otherwise said to be healthy.

The attention-enhanced model can correctly assign the side attribute (ie, maternal) for the “uncle” mentioned in the second sentence, while this could not be accomplished by the baseline model. We identified several similar cases on the test set, although these correct assignments could not be captured by the applied article level evaluation metrics.

Furthermore, we observed that the enhanced model can learn better from the implicit dispersed second-degree relative descriptions without interfering with rules created based on human knowledge. Some examples that can be correctly inferred are as follows.

The enhanced model can correctly assign the “Cousin_Paternal” tag to the children of the patient’s aunt even when the mentions are dispersed away from each other:

The **paternal aunt** died in her late 57s due to heart complications. She had five **children**. One of these children is a **daughter** who was diagnosed with breast cancer at the age of 42...

Another similar example would be the sentence, where the enhanced model can correctly determine the side and member type of the mention “son”:

Mrs. Lucas has another **paternal uncle** who has a **son** with mental retardation of unknown cause.

For the following sentence, the mentions “sisters” and “brother” within the sentence located in the later part of the document can be correctly recognized by the enhanced model as “Aunt_Paternal” and “Uncle_Paternal,” respectively:

Ms. James AJ Benjamin’s **father**, 55s, is reportedly in good health. ... He has two **sisters** and a **brother**, 63s–71s, who are reportedly in good health.

In the following description, the second mention of “mother” is successfully assigned with “Grandmother_Maternal”:

She is 5 feet 6-8 inches tall and the patient's **mother** resembles her own **mother** in facial appearance.

For the following narrative, the model learned to assign the mention “daughter” with “Sister_NA”:

The **father** has a 9-year-old **daughter** with another partner who is healthy.

We also noted that the enhanced networks can acknowledge negative clues and avoid false positive cases of observations:

She has **no** history of joint hypermobility, easy bruising, or problems with healing.

They do not look different than other members of the family, and **do not have** any major internal birth defects.

### Error Analysis

Although models with neural attentions learned to infer implicit relationships among recognized family member mentions by interpreting the contextual expressions with weighted attentions, ambiguity of the context can still occasionally confuse the model in making incorrect classifications. Some examples as such are listed.

In the following example, while the patient is Mrs. William, the attention-enhanced model focused on the terms “He,” “sister,” and “his father” and mistakenly assigned the mention “son” with the “Cousin_Paternal” tag:

... William's husband is healthy at age 38 with a history of melanoma ... He also has a 39-year-old **sister** who is healthy with a healthy 10-year-old **son**. ... His **father** is alive at age 59 with coronary disease, ...

In the following example, even with the proposed methods, the developed models could not recognize “mother’s mother’s brothers” in the second sentence as a family mention. Nevertheless, the attention-enhanced model was able to classify the first mention “brother” as the patient’s uncle and the mention “children” as the patient’s cousin. On the contrary, the baseline model classified the first and the second mentions as “brother” and “son,” respectively:

A **brother** is the father of two **children**, a male with **mental retardation** and a daughter with bicuspid mitral valve stenosis and aortic stenosis. Another of Benjamin's **mother’s mother’s brothers** is the father of two girls, one of whom ...

Based on the description, the attention-enhanced model incorrectly considered the mention “father” to be referring to the father of the patient (ie, Mrs. Henrietta):

Mrs. Henrietta is of Indian descent. The **father** of the baby is of Indonesian descent.

For the following sentence*,* the attention-enhanced model failed to ignore the in-law relationships:

Her husband has an identical twin **brother** who is healthy with fraternal twin **daughters**, ...

Some annotation errors or biases in the corpus were identified during the error analysis. First, we found that not all instances of the same family member in a given electronic health record were annotated, which means that some mentions may only be annotated once even if they refer to the same entity. In general, more cases as such occurred in the annotation of first-degree relatives rather than those of the second-degree relatives (0.586 vs 0.839) based on our estimation on the training set. One conspicuous example of this error can be found in the sentence “*The patient's **mother** is 54 now,*” where the mention “mother” was not annotated. We also noticed that the spans of some family member annotations were incorrect, which may lead to a decrease in performance. For instance, the two annotations in the sentences “*His only [**child,**] a daughter ...*” and “*This aunt has five healthy sons and one [**daughter,**] age 67, ...*” will instruct the models to accept commas to be the last token of a family mention.

### Comparison With Prior Work

Several research projects have previously worked on the FHI extraction task. Shi et al [11] developed a neural network model based on BiLSTM networks for joint learning of FHIs and the relations among them. Zhan et al [21] fine-tuned the bidirectional encoder representations from transformers [22] by including an additional Biaffine classifier adapted from the dependency parsing to extract FHIs. Most researchers considered the extraction of FHIs as a sequential labelling task and exploited sequential labelling models to address it. For instance, Kim et al [23] established an ensemble of 10 BiLSTM-CRF models along with ELMo representations to identify FHIs. Later, Wu and Verspoor [24] and Ambalavanan and Devarakonda [25] implemented similar strategies to encode the side information in their tag sets. The former applied a BiLSTM model with ELMo and a tag set that allow the model to recognize mentions of family members and determine their side information at the same time, while the latter further contained family relationship information in their tag set. Similar to this work, the attempt of these 2 works is to eliminate the application of postprocessing rules to infer the required properties of family members.

### Conclusions

In this paper, we considered the problem of FHI extraction as a sequential labelling task and presented an attention-based neural network approach to handle this problem. The main contribution of our work is that we presented an improved tag scheme that enables the model to learn and interpret the implicit relationships and side information of the recognized family members without relying on heuristic rules. Moreover, a network structure with neural attentions was proposed to exploit intrasentence and intersentence information to determine the family member mentions and side attributes requiring cross-sentence inference. The feasibility of the proposed method was assessed on the dataset released by the 2019 n2c2/OHNLP shared task on family history extraction and was officially ranked 4th among 17 teams. Although the proposed methods addressed the limitations raised, our error analysis revealed challenges including annotation bias and the requirement of common-sense reasoning, which leave room for further improvement in the future.

## Appendix

Multimedia Appendix 1 Comparison of the tag set distributions on the training set between the relation-side scheme and its enhanced version. Only the tag names within the top 10 of the distribution are shown in the figure.

## Abbreviations

A-ERS: attention-enhanced bidirectional long short-term memory-conditional random field with enhanced relation-side scheme paying attention to limited sentences
A-ERS+: attention-enhanced bidirectional long short-term memory-conditional random field with enhanced relation-side scheme paying attention to all sentences
A-RS: attention-enhanced bidirectional long short-term memory-conditional random field with relation-side scheme
B-ERS: bidirectional long short-term memory-conditional random field with enhanced relation-side scheme
BiLSTM: bidirectional long short-term memory
B-RS: bidirectional long short-term memory-conditional random field with relation-side scheme
CRF: conditional random field
ELMo: embeddings from language models
F: F score
FHI: family history information
GloVe: global vectors for word representation
IOB: inside, outside, beginning
NLP: natural language processing
P: precision
R: recall
