# Supplementary figures and images for: Family History Information Extraction With Neural Attention and an Enhanced Relation-Side Scheme: Algorithm Development and Validation
Source: JMIR Med Inform. 2020 Dec 1;8(12):e21750. doi: 10.2196/21750 (PMC7738250; doi:10.2196/21750)

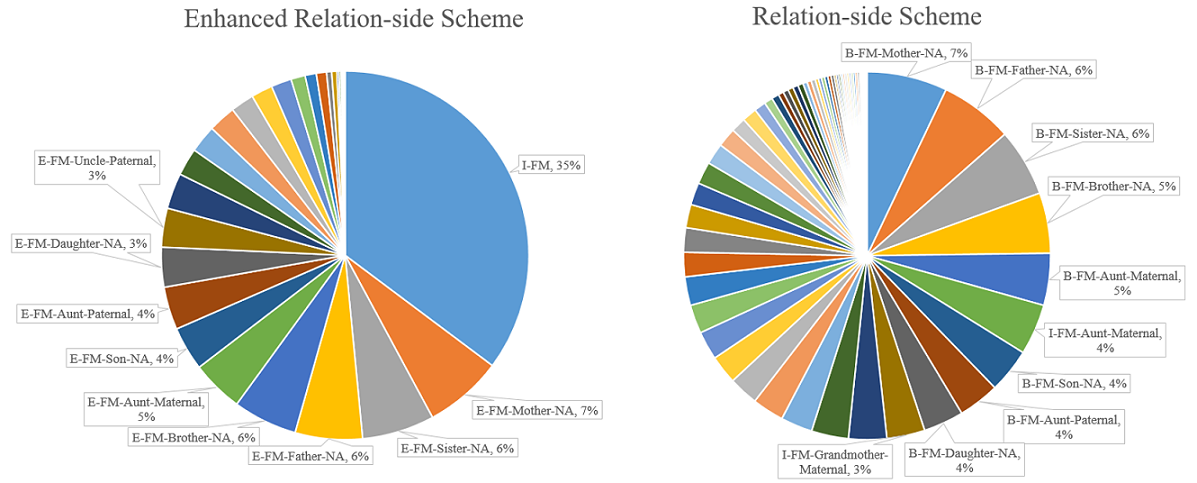

Supplement: Multimedia Appendix 1 [file medinform_v8i12e21750_app1.png]
